# Prognostic value of sarcopenia in patients with rectal cancer: A meta-analysis

**DOI:** 10.1371/journal.pone.0270332

**Published:** 2022-06-24

**Authors:** Yueli Zhu, Xiaoming Guo, Qin Zhang, Yunmei Yang

**Affiliations:** 1 Department of Geriatrics, The First Affiliated Hospital, School of Medicine, Zhejiang University, Hangzhou, China; 2 Department of Neurosurgery, Tongde Hospital of Zhejiang Province, Hangzhou, China; Shuguang Hospital, CHINA

## Abstract

**Background:**

Sarcopenia is usually characterized by the loss of skeletal muscle mass and impaired muscle function which is commonly seen in the elderly. It has been found to be associated with poorer prognoses in many types of cancer. Computed tomography (CT) scan is frequently used to assess skeletal muscle mass and further calculate skeletal muscle index (SMI) at the third lumbar vertebra level (L3), which is used to define sarcopenia. The purpose of this meta-analysis was to assess the prognostic value of sarcopenia for overall survival (OS) in patients with rectal cancer.

**Methods:**

We performed a systematic search to find relevant studies published up to 14 January 2021 in PubMed, Embase, Web of science and Scopus. In our meta-analysis, studies comparing OS in rectal cancer patients with sarcopenia versus those without were included. Quality assessment for included studies was evaluated according to the Quality in Prognosis Studies (QUIPS) tool. We directly extracted hazard ratios (HRs) with 95% confidence intervals (CIs) in both univariate and multivariate analyses from each study. The Cochrane Collaboration’s Review Manager 5.4 software was applied to analyze data. The quality of evidence was assessed using the Grading of Recommendations Assessment, Development, and Evaluation (GRADE) guidelines and website GRADEpro.

**Results:**

Finally, a total of 7 studies involving 2377 patients were included. The pooled HRs were 2.10 (95% CI 1.33–3.32, *P* = 0.001) and 2.37 (95% CI 1.13–4.98, *P* = 0.02) using random-effects models in univariate and multivariate analyses, respectively. The results showed a significant association between sarcopenia and OS in patients with rectal cancer. The quality of the evidence for OS was moderate for both univariate and multivariate analyses.

**Conclusion:**

CT-defined sarcopenia is an independent predictor for worse OS in patients with rectal cancer. Future studies with a more stringent definition of sarcopenia are required to further confirm our findings.

## Introduction

Colorectal cancer, with rectal cancer accounting for approximately 30%, is the third most common form of cancer and ranks second in terms of mortality worldwide [1, 2]. And rectal cancer has been a challenging disease to treat due to the anatomical structure and also due to a high rate of local recurrence. At present, the combination of total mesorectal excision (TME) and neoadjuvant chemoradiotherapy (nCRT) has been the standard treatment for patients with locally advanced rectal cancer. And it has been reported that preoperative CRT can improve local control of rectal cancer and reduce toxicity without improving overall survival (OS) compared with postoperative CRT [3]. At present, identifying modifiable risk factors associated with the prognoses of patients with rectal cancer is of great value.

As we know, cancer patients are going through changes in their body composition along with the disease process. According to the report of the European Working Group on Sarcopenia in Older People (EWGSOP), sarcopenia can be defined as low muscle mass and impaired muscle function (strength or performance) [4]. Sarcopenia is common in older adults and has been found to be associated with poorer prognoses in many types of cancer, including head and neck cancer, lung cancer, breast cancer, and so on [5–7]. The third lumbar vertebrae skeletal muscle index (L3 SMI) is often calculated based on a computed tomography (CT) scan to define sarcopenia [8]. For cancer patients, CT scans are routinely performed for assessing tumor lesions and monitoring tumor metastasis, and it has been a common approach to measure skeletal muscle mass without extra radiation.

Currently, there is an increasing focus on the relationship between sarcopenia and cancer outcomes. Previous meta-analyses have found that sarcopenia is a negative predictor for OS in patients with colorectal cancer [9, 10]. Several studies have investigated the association between sarcopenia and the OS in patients with rectal cancer, but the result remains controversial. So far, the prognostic value of sarcopenia in rectal cancer has not been systematically evaluated yet.

Here, we performed a meta-analysis aiming to explore whether sarcopenia defined by L3 SMI can negatively influence OS among rectal cancer patients.

## Methods

This meta-analysis study was conducted according to the Preferred Reporting Items for Systematic Reviews and Meta-Analyses (PRISMA) guidelines (S1 Table) [11].

### Search strategy

Two reviewers (Y.Z. and X.G.) independently carried out a systematic literature search to identify relevant studies. And disagreements were solved by discussion with a third reviewer if necessary (Q.Z.). This study was conducted according to a pre-designed protocol. PubMed, Embase, Web of science and Scopus were all screened from their inception to 14 January 2021. A combination of medical subject headings (MeSH) terms and keywords were applied relating to rectum, cancer and sarcopenia. The full search strategy was presented in S1 File. Besides, the references of included studies and relevant reviews were manually searched in order to detect additional eligible studies.

### Eligibility criteria

Studies were included in this meta-analysis if they met all of the following criteria: (1) studies of patients with rectal cancer; (2) studies that compared OS between patients with sarcopenia versus those without; (3) skeletal muscle mass measured by CT quantificationally at the level of L3; (4) studies that reported hazard ratios (HRs) and 95% confidence intervals (CIs) for OS. Case reports, reviews, comments, conference abstracts, and animal experiments were excluded. Furthermore, we excluded studies that did not provide valid data, such as HRs and 95% CIs.

### Data extraction

Two reviewers (Y.Z. and X.G.) independently extracted relevant data from all included studies using a standardized form. Disagreements were resolved through consultation with a third reviewer (Q.Z.). For each study, the following details were extracted: first author’s name, year of publication, country, study design, number of patients, mean/median population age, the percentage of male, disease stage, SMI measurement, cut-off points of SMI, prevalence of sarcopenia, and HRs with 95%CIs for OS. And we extracted HRs with corresponding 95% CIs in univariate and multivariate analyses from each study if data was provided.

### Quality assessment

Quality assessment for included studies was performed by two independent reviewers (Y.Z. and X.G.) according to the Quality in Prognosis Studies (QUIPS) tool [12, 13]. This tool consists of six bias domains: study participation, study attrition, prognostic factor measurement, outcome measurement, study confounding, and statistical analysis and reporting. Each of these domains is categorized as high, moderate, or low risk based on several criteria. The study with ≤2 domains of moderate risk of bias and all others of low risk of bias is rated as having a low risk of bias. The study with >2 domains of moderate risk of bias and all others of low risk of bias is rated as having a moderate risk of bias. The study with high risk of bias in at least one domain is rated as having a high risk of bias. Any discrepancies were resolved through discussion with a third reviewer (Q.Z.). We further assessed the quality of evidence for the outcomes in our meta-analysis using the Grading of Recommendations Assessment, Development, and Evaluation (GRADE) guidelines and website GRADEpro [14]. For each outcome, some variables were imputed to automatically rate the quality of evidence, including type of study design, risk of bias, inconsistency, indirectness, imprecision, and other considerations. And the outcome was rated as four levels: high, moderate, low or very low based on GRADE guidelines.

### Statistical analysis

We performed this meta-analysis by using Review Manager 5.4 software (The Cochrane Collaboration). HRs and 95% CIs of OS associated with sarcopenia were directly retrieved from the included studies. Heterogeneity between studies was estimated by performing the chi-square test and the *I*^*2*^ statistic. *P* < 0.1 and *I*^*2*^ > 50% were used to define statistically significant heterogeneity between studies. In view of the different cut-off points of SMI for the diagnosis of sarcopenia, there may be different effect sizes among different studies and the between-study heterogeneity was expected to be at least moderate. Therefore, random-effects models were applied for both univariate and multivariate data in our study [15]. A Tau^2^ value was produced to estimate the between-study variance in a random-effects meta-analysis. Besides, we performed sensitivity analyses to investigate the sources of heterogeneity. And funnel plots were used to identify potential publication bias. The symmetric distribution of funnel plot shapes suggested that the publication bias was low. *P* < 0.05 was considered to be statistically significant.

## Results

### Search results/included studies

**Fig 1:**
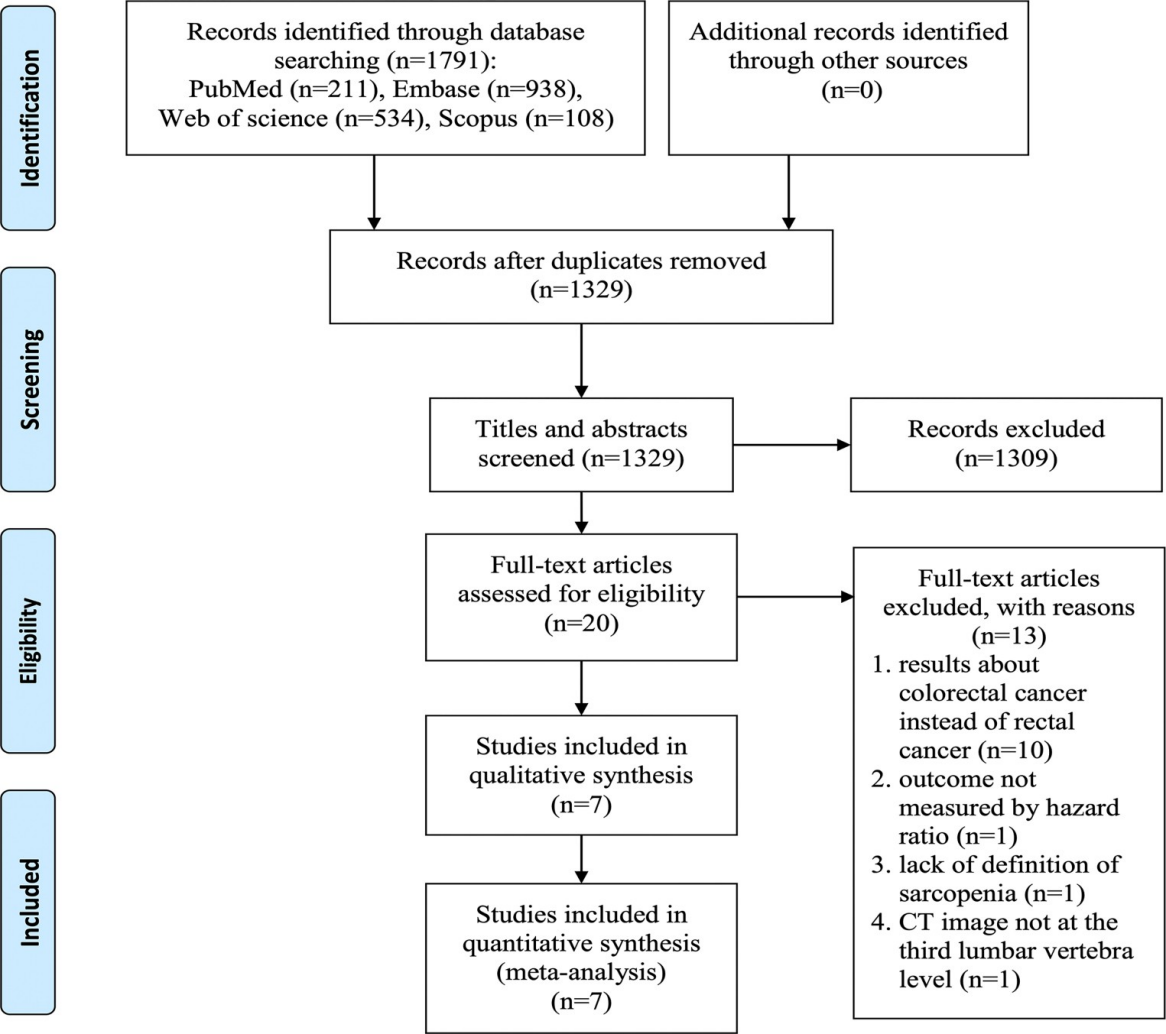
Flow chart of the selection process for included studies.

The literature searches of four databases yielded a total of 1791 potential relevant records. After removing 462 duplicates, titles and abstracts were screened in 1329 studies. Among these, 20 studies underwent full text review. Finally, 7 studies were identified as suitable for this meta-analysis [16–22]. The detailed flow diagram of the selection process was shown in Fig 1.

### Patient characteristics

Ultimately, 7 retrospective studies were included, comprising a total of 2377 patients. All these included studies evaluated L3 SMI for diagnosing sarcopenia and its association with OS in patients with rectal cancer. L3 SMI was the variable that calculated as the total muscle area at L3 the level (cm^2^) divided by height square (m^2^) based on axial CT scan. One study involving patients with colorectal cancer was included, since the outcome data about patients with rectal cancer had been provided in the article [20]. In most of the included studies, sarcopenia was diagnosed according to SMI by CT images taken before nCRT. And in two studies, the outcome data about sarcopenia diagnosed by CT scans measured before and after nCRT were both obtainable [18, 21], and we selected the data with CT scans taken before nCRT. The diagnosis of rectal cancer was confirmed in all included patients, mostly between stages II–III. And most of these rectal cancer patients underwent nCRT combined with surgery. Study characteristics were displayed in Table 1.

**Table 1:** Study characteristics of included studies.

Study	Country	Study design	Number of cases	Age^a^	Male (%)	Disease stage	Sarcopenia measurement and definitions		Prevalence of sarcopenia (%)	Outcome data reported
							Measurement	Cut-off points of SMI (cm^2^ /m^2^)		
Choi et al., 2017 [16]	South Korea	Retrospective	188	61.3	62.2	II-III	CT image at the 3rd lumbar level	Men: 52.4	39.4	OS^b^
								Women: 38.5		
Chung et al., 2019 [21]	South Korea	Retrospective	93	NR	64.5	II-III	CT image at the 3rd lumbar level	Men: 52.4	NR	OS^e^
								Women: 38.5		
Han et al., 2020 [22]	South Korea	Retrospective	1384	59.0	64.2	0-III	CT image at the 3rd lumbar level	Men: 52.4	68.2	OS^b^
								Women: 38.5		
Hopkins et al., 2019 [20]	Canada	Retrospective	381	NR	69.6	I-III	CT image at the 3rd lumbar level	Men: 43 or 53^c^	NR	OS^d^
								Women: 41		
Levolger et al., 2017 [18]	Netherlands	Retrospective	122	61.0	58.2	II-IV	CT image at the 3rd lumbar level	Men: 52.4	NR	OS^e^
								Women: 38.5		
Park et al., 2018 [19]	South Korea	Retrospective	65	71.0	70.8	II-III	CT image at the 3rd lumbar level	Men: 49	38.5	OS^b^
								Women: 31		
Takeda et al., 2018 [17]	Japan	Retrospective	144	NR	70.8	II-III	CT image at the 3rd lumbar level	Men: 45	25.7	OS^b^
								Women: 33.8		

SMI, skeletal muscle index; OS, overall survival; NR, not reported.

^a^Mean or median age as reported.

^b^Univariate and multivariate data.

^c^SMI < 43 cm^2^ /m^2^ when body mass index < 25 kg/m^2^ or SMI < 53 cm^2^ /m^2^ when body mass index ≥ 25 kg/m^2^.

^d^Multivariate data.

^e^Univariate data.

### Risk of bias and GRADE assessment

The methodological quality of all included 7 studies were assessed by using the QUIPS tool on six domains. For the study participation domain, the risk of bias was moderate in four studies [17, 19–21]. And a moderate risk of bias was ranked in all included studies of study attrition [16–22], one study of prognostic factor measurement [17], and one study of outcome measurement [18]. Regarding study confounding, two studies were ranked with a high risk of bias [18, 19]. To sum up, four studies were judged as having an overall low risk of bias [16, 20–22], one study was at an overall moderate risk of bias [17], and the other two studies were assessed as being at an overall high risk of bias [18, 19] (Table 2). According to GRADEpro tool, the quality of evidence was downgraded for outcomes due to risk of bias and inconsistency. And the quality of evidence was moderate for both univariate and multivariate data with respect to the association between sarcopenia and OS (Table 3).

**Table 2:** Risk of bias for the included studies assessed by the Quality in Prognosis Studies (QUIPS) tool.

	Study participation	Study attrition	Prognostic factor measurement	Outcome measurement	Study confounding	Statistical analysis and reporting	Overall risk of bias
Choi et al. [16]	Low	Moderate	Low	Low	Low	Low	Low
Chung et al. [21]	Moderate	Moderate	Low	Low	Low	Low	Low
Han et al. [22]	Low	Moderate	Low	Low	Low	Low	Low
Hopkins et al. [20]	Moderate	Moderate	Low	Low	Low	Low	Low
Levolger et al. [18]	Low	Moderate	Low	Moderate	High	Low	High
Park et al. [19]	Moderate	Moderate	Low	Low	High	Low	High
Takeda et al. [17]	Moderate	Moderate	Moderate	Low	Low	Low	Moderate

**Table 3:** GRADE evidence profile: Sarcopenia vs. non-sarcopenia for OS in rectal cancer patients.

Certainty assessment							No. of patients	Effect	Certainty	Importance
No. of studies	Study design	Risk of bias	Inconsistency	Indirectness	Imprecision	Other considerations		Relative (95% CI)		
Overall survival- univariate data (n = 6)	non-randomised studies	serious	serious	not serious	not serious	strong association	1996	HR 2.10 (1.33–3.32)	⨁⨁⨁◯ MODERATE	CRITICAL
Overall survival- multivariate data (n = 5)	non-randomised studies	serious	serious	not serious	not serious	strong association	2162	HR 2.37 (1.13–4.98)	⨁⨁⨁◯ MODERATE	CRITICAL

### Sarcopenia and OS in rectal cancer

**Fig 2:**
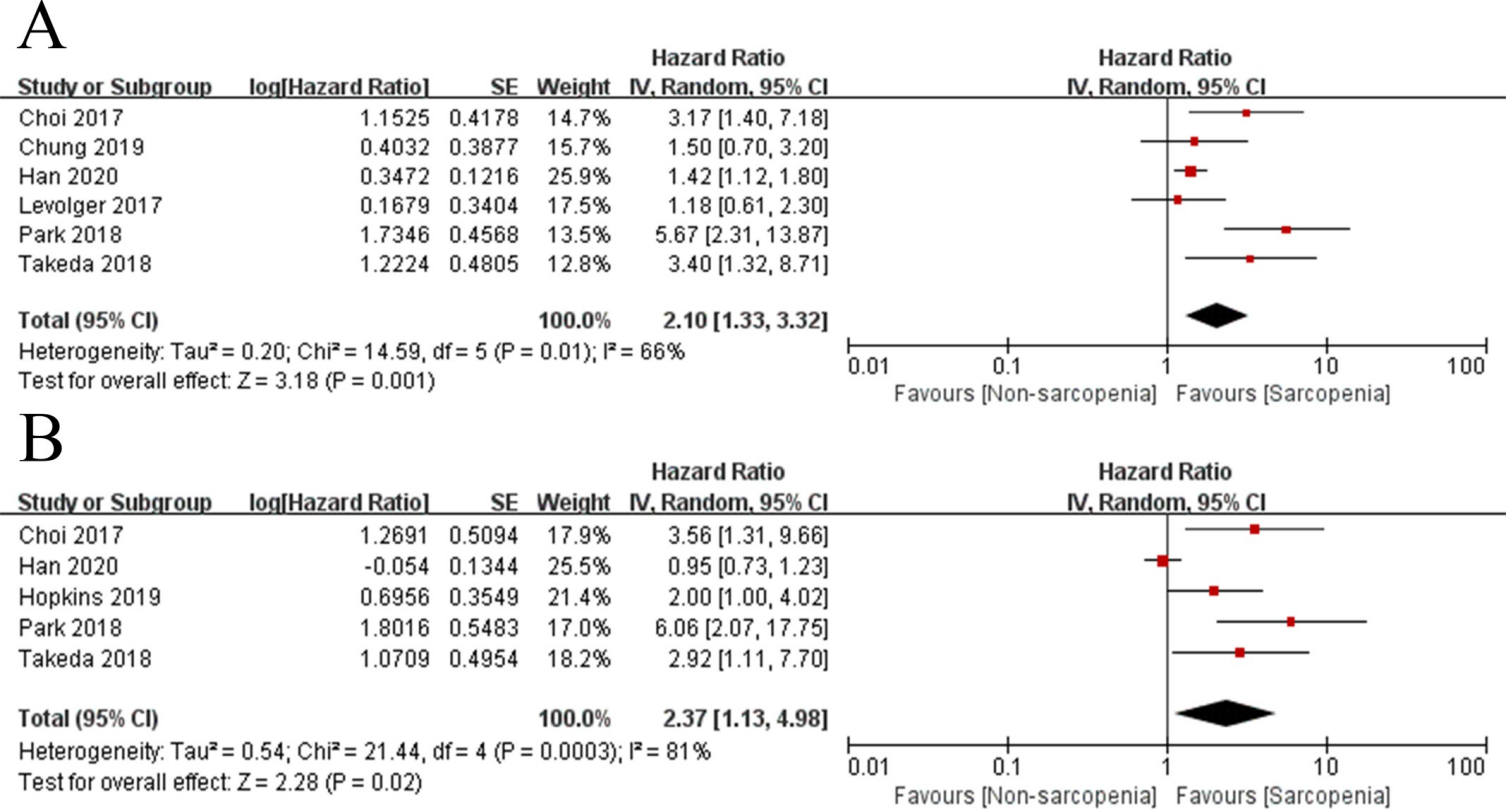
Forest plots of hazard ratios for overall survival in rectal cancer patients with sarcopenia versus those without. (A) univariate data, (B) multivariate data.

The impact of sarcopenia on OS was described in Fig 2, including univariate and multivariate analyses. As for univariate data, the random-effects model was used due to the existence of heterogeneity between studies (Tau^2^ = 0.20, Chi^2^ = 14.59, *P* = 0.01, *I*^*2*^ = 66%). And the pooled analysis showed that there was a significant association between sarcopenia and poorer OS in rectal cancer patients (HR 2.10, 95% CI 1.33–3.32, *P* = 0.001) (Fig 2A). With regard to multivariate data, the outcome using a random-effects model indicated that sarcopenia was negatively associated with OS (HR 2.37, 95% CI 1.13–4.98, *P* = 0.02), in spite of obvious heterogeneity (Tau^2^ = 0.54, Chi^2^ = 21.44, *P* = 0.0003, *I*^*2*^ = 81%) (Fig 2B).

### Sensitivity analysis and publication bias

There were seven studies in our meta-analysis to investigate the association between sarcopenia and OS. For both univariate and multivariate analyses, the significant heterogeneity between studies was found. Regarding univariate data, the heterogeneity among studies decreased to 41% when we excluded the study conducted by Park et al. [19]. As for multivariate data, when we removed the study carried out by Han et al. [22], the heterogeneity dramatically decreased from 81% to 1%.

**Fig 3:**
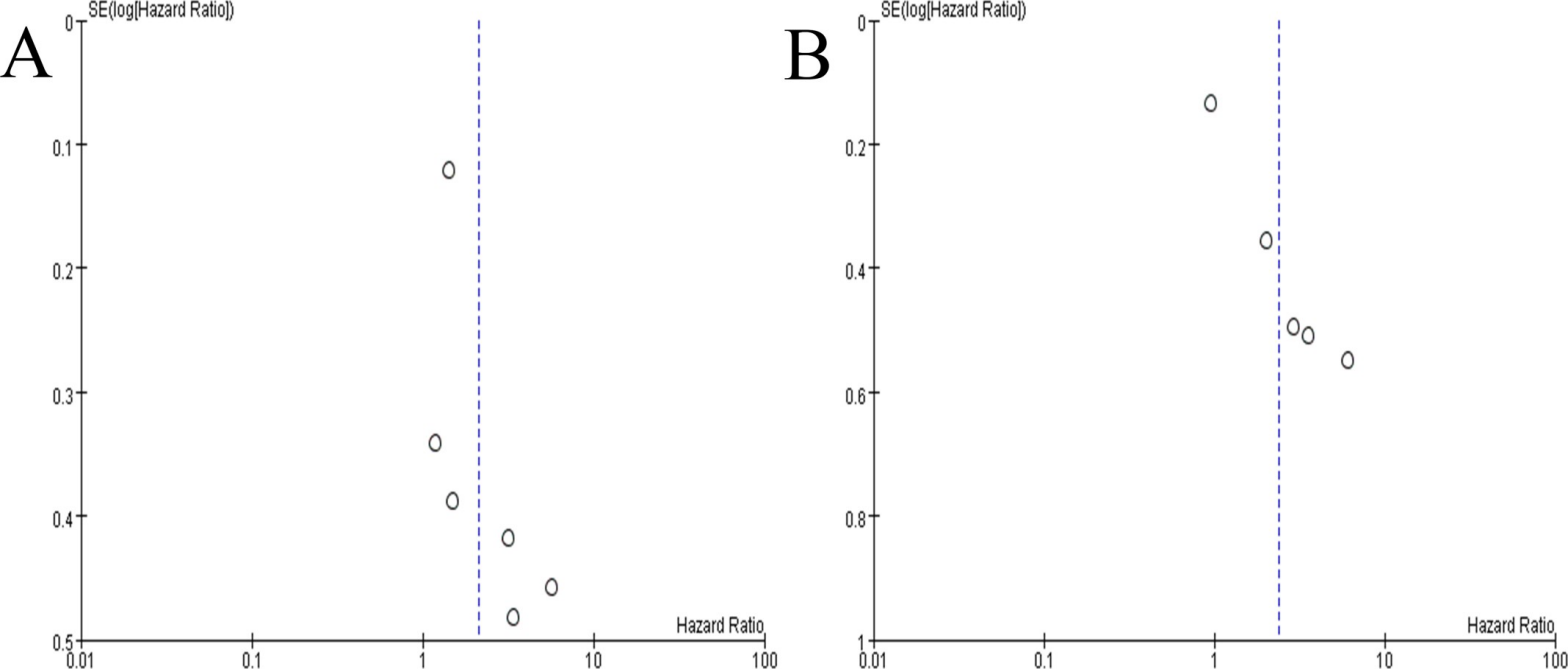
Funnel plots for assessing publication bias. (A) univariate data, (B) multivariate data.

The publication bias was detected by funnel plots. And the funnel plots revealed a symmetric distribution (Fig 3), indicating the absence of publication bias among these included studies.

## Discussion

This meta-analysis demonstrated that sarcopenia was associated with worse OS in patients with rectal cancer of both the univariate and multivariate data. It was the first meta-analysis to investigate the relationship between sarcopenia and OS in rectal cancer patients, and the result was in line with most previous observational studies. The quality of evidence for the outcome was considered moderate for both univariate and multivariate analyses on the basis of the GRADE rating.

Sarcopenia, which is defined by the age-related loss of skeletal muscle mass and impaired muscle function, has been increasingly investigated for the predictive value in cancer patients over the past decade [4]. Sarcopenia should not be disregarded in clinical work, since the incidence among geriatric cancer patients varied from 12.5% to 57.7% [23]. And cancer has been a major cause of secondary sarcopenia [24]. In current cancer studies, the diagnosis of sarcopenia depended mainly on the low SMI, without assessing muscle strength and physical performance. And further studies with a more comprehensive definition of sarcopenia are needed to reach more reliable conclusions. CT scan is able to be a practical method to assess body composition on account of the routine use for clinical staging in cancer patients. The main purpose of this meta-analysis was to explore the effects of sarcopenia on the prognosis of rectal cancer, as the current findings of studies had not been summarized so far. In our meta-analysis, sarcopenia was diagnosed with SMI at the level of L3 by CT scan in all seven included studies, which was the gold standard of measurement. And we believe it can decease the heterogeneity and help to reach a convincing conclusion.

As a result, the incidence rate of sarcopenia among rectal cancer patients had a range from 25.7 to 68.2%. The rate seemed to be relatively high in view of the mean age (about 60 years) and the small proportion of patients with metastatic disease. And it may be due to the usage of common sex-specific cut-off points of SMI for sarcopenia in some included studies from South Korea [16, 21, 22], rather than using Korean-specific cut-off points (49 cm^2^/m^2^ for men and 31 cm^2^/m^2^ for women) [25]. And significant differences were observed in OS between patients with sarcopenia and those without in both univariate (HR 2.10, 95% CI 1.33–3.32, *P* = 0.001) and multivariate analyses (HR 2.37, 95% CI 1.13–4.98, *P* = 0.02) using random-effects models. The pooled HR was even higher in the multivariate analysis. Based on these results, sarcopenia was demonstrated to be significantly associated with shorter survival in patients with rectal cancer. Of note, we had tried to use the data of sarcopenia defined by pre-treatment CT. This said, sarcopenia was assessed before the treatment for rectal cancer. In light of our findings, screening pre-treatment sarcopenia exhibited significance among cancer patients because pre-treatment sarcopenia was independently related to the poorer survival. Thus, timely detection and intervention of sarcopenia before cancer treatment should contribute to improve patient outcomes.

We found obvious heterogeneity in both univariate and multivariate analyses. And sensitivity analyses were performed in order to explore the potential source of heterogeneity. When we removed the study conducted by Park et al. [19] in univariate analysis, the heterogeneity decreased obviously and the result was not influenced. The cut-off point of SMI to define sarcopenia in the study by Park et al. was lower than that in other studies. For this reason, sarcopenia might be more severe in the sarcopenia group of this study than those in other studies, which further magnified the negative impact of sarcopenia on OS. Thus, the study by Park et al. might be the source of heterogeneity. As for multivariate analysis, the heterogeneity decreased dramatically after removal of the study conducted by Han et al. [22] and the result was consistent with the outcome pooled by all studies. Perhaps, it can be attributed to the usage of common cut-off points of SMI for sarcopenia instead of using Korean-specific cut-off points in this study, which may increase the incidence rate of sarcopenia and accordingly reduce the negative effects of sarcopenia on OS.

To date, the reason why sarcopenia is negatively associated with OS in rectal cancer patients is still unclear. It may be explained by systemic inflammation. Sarcopenia reflects a state of increased catabolism and is related to a stronger postoperative inflammatory response in colorectal cancer [26, 27]. Systemic inflammation can be an independent factor for poorer outcomes in patients with rectal cancer [28]. And the co-existence of inflammation and sarcopenia leads to a high mortality risk [29]. The exact mechanisms need to be investigated further.

There are still several limitations in our meta-analysis. Firstly, sarcopenia was defined using cut-off points of SMI to meet the criterion of low muscle mass. However, the assessments of muscle strength and physical performance were not included in all these studies, which should be considered according to the diagnostic criteria for sarcopenia by the EWGSOP [4]. And the cut-off points of SMI varied among studies. Secondly, two of these included studies had high risk of bias, while other studies were classified as low to moderate risk of bias. Finally, only seven studies were included in this meta-analysis and all of them were retrospective in nature.

## Conclusion

In general, we make the conclusion that sarcopenia is independently associated with worse OS in patients with rectal cancer. Future studies with a comprehensive definition of sarcopenia and different type of designs are needed to strengthen the evidence and further confirm our conclusion. We suppose that appropriate nutrition supplement and strengthening exercise may contribute to improve the prognosis of patients with rectal cancer, which can be verified in further researches.

## Supporting information

S1 File Search strategy. (DOCX)

S1 Table PRISMA checklist. (DOC)
