# Supplementary material for: Prognostic value of sarcopenia in patients with rectal cancer: A meta-analysis
Source: PLoS One. 2022 Jun 24;17(6):e0270332. doi: 10.1371/journal.pone.0270332 (PMC9231737; doi:10.1371/journal.pone.0270332)
Supplement: S1 File — (DOCX) [file pone.0270332.s001.docx]

**S1 File**

**Search Terms**

1. sarcopenia; muscle wasting; muscle loss; muscle depletion; muscle atrophy; low muscle mass
2. rectal; rectum
3. cancer*; carcinoma*; neoplasm*; tumor*; malignan*

1 AND (2 AND 3)

**Pubmed: 211**

(sarcopenia OR muscle wasting OR muscle loss OR muscle depletion OR muscle atrophy OR low muscle mass) AND (rectal OR rectum) AND (cancer* OR carcinoma* OR neoplasm* OR tumor* OR malignan*)

**Embase: 938**

('sarcopenia'/exp OR sarcopenia OR 'muscle wasting'/exp OR 'muscle wasting' OR (('muscle'/exp OR muscle) AND wasting) OR 'muscle loss'/exp OR 'muscle loss' OR (('muscle'/exp OR muscle) AND ('loss'/exp OR loss)) OR 'muscle depletion' OR (('muscle'/exp OR muscle) AND ('depletion'/exp OR depletion)) OR 'muscle atrophy'/exp OR 'muscle atrophy' OR (('muscle'/exp OR muscle) AND ('atrophy'/exp OR atrophy)) OR 'low muscle mass'/exp OR 'low muscle mass' OR (low AND ('muscle'/exp OR muscle) AND ('mass'/exp OR mass))) AND (rectal OR 'rectum'/exp OR rectum) AND (cancer* OR carcinoma* OR neoplasm* OR tumor* OR malignan*)

**Web of science: 534**

((sarcopenia OR muscle wasting OR muscle loss OR muscle depletion OR muscle atrophy OR low muscle mass) AND (rectal OR rectum) AND (cancer* OR carcinoma* OR neoplasm* OR tumor* OR malignan*))

**Scopus: 108**

TITLE-ABS-KEY ((sarcopenia OR muscle AND wasting OR muscle AND loss OR muscle AND depletion OR muscle AND atrophy OR low AND muscle AND mass) AND (rectal OR rectum) AND (cancer* OR carcinoma* OR neoplasm* OR tumor* OR malignan*))
